# The Story of the Insane from Year to Year

**Published:** 1899-01-28

**Authors:** 


### THE STORY OF THE INSANE FROM YEAR TO YEAR.

(Eleventh Series.)

### WORCESTER COUNTY ASYLUM.

An increase from 1,043 to 1,099 has to be noted here. There were 179 first admissions, 30 readmissions, 63 recoveries, 10 discharged relieved, and 75 deaths. These numbers show that the percentage of recoveries on admissions was 32·3, and that of deaths was 7·0 on the average number resident. Twenty-five of the deaths were caused by cerebral and spinal diseases, 15 by consumption, 4 by pneumonia, 1 by enteritis, and in 2 others dysentery was part cause. Of the year's admissions 26 were driven insane by drink, 30 by hereditary tendency, 52 by previous attacks, and 17 by congenital defect. During the year the asylum contained about 40 more patients than it could properly accommodate, but there were 39 private patients in residence. Like so many other asylums this was visited by dysentery, and Dr. Hartnell thinks he has adopted means of checking its progress by the use of chlorinated lime. We hope it may prove so, but we believe this system has been tried before. However, if it be proved, after extended trial, successful at Worcester, it would show that the plan had not been so carefully carried out in other asylums. Dr. Hartnell takes the view of minority, and holds that insanity is increasing. It must ever be very difficult to obtain data sufficiently accurate to enable anyone to arrive at a perfectly trustworthy conclusion on this subject, but at present we are inclined to agree with Dr. Hartnell. Dr. Cooke, the former medical superintendent, has been appointed a Commissioner in Lunacy. When alluding to this, the visitors say: "We cannot more fitly express our sense of the loss which we are shortly to attain than by repeating the words of a resolution at which we unanimously arrived when the fact of Dr. Cooke's appointment was made known to us." This resolution expresses the deep regret of the committee on receiving the resignation, and conveys hearty congratulations to Dr. Cooke on obtaining a seat on the Board of Lunacy. We join in these congratulations, although we think Dr. Cooke would have been better at Worcester. The weekly rate of maintenance is 8s. 2d. per head.

### PLYMOUTH BOROUGH ASYLUM.

This is one of the smallest asylums in England, and from this, its sixth annual report, we learn that it contained only 249 patients on the last day of the year 1897. There were 92 first admissions, 6 readmissions, 23 recoveries, and 26 deaths. Calculated on the admissions the recoveries stand at 27·05 per cent., but this is considerably less than their average, which for the six years stands at 48·63. The deaths, calculated on the average number resident, stand at 10·27, which is somewhat above their average, and also a little higher than in county asylums generally. Various moral causes account for 18 of the year's admissions, intemperance in drink for 6, hereditary influences for 15, and previous attacks 19, while 132 are stated to be unknown. This does not seem quite clear, as there were only 98 admissions. Of the deaths 9 were caused by general paralysis, 3 by heart disease, and 1 by consumption. An increase in the number of beds is spoken of, and as the administrative departments are large enough to deal with considerable additions, there can be no doubt as to propriety of anticipating the wants of the borough. Nothing is so extravagant in asylum management as procrastination. Dr. Davis gives good reason for immediate action, and it is gratifying to notice that the Committee of Visitors were, on the date of this report, considering plans for an additional 200 beds. Dr. Davis and his colleague, Dr. Bowes, conduct classes for training attendants and nurses in their duties, and with a view to their passing the examination for the nursing certificate; and we are informed that no one from Plymouth has failed to pass the examination with credit. If this be the examination of the Medico-Psychological Association, we hope that Dr. Davis will supplement it by a longer and higher training, and so place his staff at least on a level with the trained nurses in general hospitals. The visitors express their acknowledgments to Dr. Davis and his colleagues "for the high ability they have displayed in their respective offices." The weekly rate of maintenance is 12s. 0¾d.

